# New Curculionoidea records from New Brunswick, Canada with an addition to the fauna of Nova Scotia

**DOI:** 10.3897/zookeys.573.7444

**Published:** 2016-03-24

**Authors:** Reginald P. Webster, Robert S. Anderson, Vincent L. Webster, Chantelle A. Alderson, Cory C. Hughes, Jon D. Sweeney

**Affiliations:** 1 24 Mill Stream Drive, Charters Settlement, NB, Canada E3C 1X1; 2 Research Division, Canadian Museum of Nature, P.O. Box 3443, Station D, Ottawa, ON, Canada K1P 6P4; 3 Natural Resources Canada, Canadian Forest Service - Atlantic Forestry Centre, 1350 Regent St., P.O. Box 4000, Fredericton, NB, Canada E3B 5P7

**Keywords:** Anthribidae, Nemonychidae, Brentidae, Brachyceridae, Curculionidae, new records, Canada, New Brunswick, Nova Scotia

## Abstract

This paper presents 27 new records of Curculionoidea for the province of New Brunswick, Canada, including three species new to Canada, and 12 adventive species, as follows: *Eusphryrus walshii* LeConte, *Choragus harrisii* LeConte (newly recorded for Canada), *Choragus zimmermanni* LeConte (newly recorded for Canada) (Anthribidae); *Cimberis pallipennis* (Blatchley) (Nemonychidae); *Nanophyes marmoratus marmoratus* (Goeze) (Brentidae); *Procas lecontei* Bedel (Brachyceridae); *Anthonomus pusillus* LeConte (newly recorded for Canada), Anthonomus (Cnemocyllus) pictus Blatchley, *Archarius salicivorus* (Paykull), *Dorytomus hirtus* LeConte, *Ellescus bipunctatus* (Linnaeus), *Mecinus janthinus* (Germar), *Myrmex chevrolatii* (Horn), *Madarellus undulatus* (Say), *Microplontus campestris* (Gyllenhal), *Pelenomus waltoni* (Boheman), *Rhinoncus bruchoides* (Herbst), *Rhinoncus perpendicularis* (Reich), *Cossonus impressifrons* Boheman, *Cossonus pacificus* Van Dyke, *Rhyncolus knowltoni* (Thatcher), *Eubulus bisignatus* (Say), *Polydrusus cervinus* (Linnaeus), *Magdalis piceae* Buchanan, *Procryphalus mucronatus* (LeConte), *Ips grandicollis* (Eichhoff), and *Xyleborinus attenuatus* (Blandford). Recent name changes in the genus *Rhinoncus* are applied to species known from New Brunswick. In addition, *Orchestes alni* (Linnaeus) is newly recorded from Nova Scotia.

## Introduction

The Curculionidae of New Brunswick were first reviewed by Majka et al. (2007), adding 77 species to the faunal list of the province. Later, Webster et al. (2012) newly recorded three species of Anthribidae, four Brentidae, three Dryophthoridae, three Brachyceridae, and 50 species of Curculionidae. Shortly after this, another four species of Anthribidae, one Brentidae and 11 species of Curculionidae were added to the faunal list of New Brunswick by Douglas et al. (2013). Cognato et al. (2015) reported the occurrence of *Dryocoetes kriviolutzkajae* Mandelshtam in New Brunswick. It is unclear whether this is an introduction from Russia or a Holarctic species (Cognato et al. 2015). Since 2013, 27 additional species of Curculionoidea from the families Anthribidae, Nemonychidae, Brentidae, Brachyceridae, and Curculionidae have been documented for New Brunswick, including three species new to Canada. Twelve of these are adventive species. One species is also newly reported from Nova Scotia. The purpose of this paper is to report on these new records.

## Methods and conventions

**Collection methods.** Specimens were collected by sweeping vegetation in various habitats and from Lindgren 12-funnel trap samples during a study to develop improved tools for the detection of invasive species of Cerambycidae. These traps are visually similar to tree trunks and are often effective for sampling species of Coleoptera that live in microhabitats associated with standing trees (Lindgren 1983). In many sites, equal numbers of traps were deployed in the canopy and 1 m high under trees. See Webster et al. (2012) and Hughes et al. (2014) for details of the methods used to deploy Lindgren traps and for sample collection.

A description of the habitat was recorded for all specimens collected during this survey. Locality and habitat data are presented as on labels for each record. Two labels were used on many specimens, one that included the locality, collection date, and collector, and one with macro- and microhabitat data and collection method. Information from the two labels is separated by a // in the data presented from each specimen.

**Distribution.** Every species is cited with current distribution in Canada and Alaska, using abbreviations for the state, provinces, and territories. New records for New Brunswick are indicated in **bold** under **Distribution in Canada and Alaska**. The following abbreviations are used in the text:

AK Alaska


MB Manitoba


YT Yukon Territory


ON Ontario


NT Northwest Territories


QC Quebec


NU Nunavut


NB New Brunswick


BC British Columbia


PE Prince Edward Island


AB Alberta


NS Nova Scotia


SK Saskatchewan


NF & LB Newfoundland and Labrador*


*Newfoundland and Labrador are each treated separately under the current Distribution in Canada and Alaska.

USA state abbreviations follow those of the US Postal Service. Acronyms of collections examined or where specimens reside referred to in this study are as follows:

AFC
 Atlantic Forestry Centre, Fredericton, New Brunswick, Canada 


CMNC
Canadian Museum of Nature, Ottawa, Ontario, Canada 


NBM
 New Brunswick Museum, Saint John, New Brunswick, Canada 


RWC
 Reginald P. Webster Collection, Charters Settlement, New Brunswick, Canada 


## Results

We newly report on 28 species of Curculionoidea, including three new Canadian records, in the families Anthribidae (3), Nemonychidae (1), Brentidae (1), Brachyceridae (1), and Curculionidae (22), including one species of Curculionidae new to Nova Scotia. Twenty-four of the 28 species reported in this study were captured in Lindgren 12-funnel traps; 18 were collected only in these traps. Four species were collected by sweeping foliage, and one was found under bark.

### Species accounts

Species with a † are adventive to Canada. The determination that a species was a new record is based on information in the print version of Bousquet et al. (2013). The classification used below follows Bouchard et al. (2011).

### Family Anthribidae Billberg, 1820

#### Subfamily Anthribinae Billberg, 1820

##### Tribe Zygaenodini Lacordaire, 1865

###### Eusphyrus walshii

Taxon classificationAnimaliaColeopteraAnthribidae

LeConte, 1876

####### Material examined.

**New Brunswick, York Co.**, Fredericton, Odell Park, 45.9439°N, 66.6666°W, 24.VI-9.VII.2013, 7-19.VIII.2013, C. Alderson & V. Webster // Hardwood stand, Lindgren funnel traps in canopy (2, RWC); same locality, forest type, and collection method but 19.VIII-5.IX.2013 (1, RWC); same locality and forest type but 45.9508°N, 66.6723°W, 14-28.VII.2015, 10-25.VIII.2015, 25.VIII-9.IX.2015, C. Alderson & V. Webster // Lindgren funnel traps in canopy (2 AFC; 5, RWC); Keswick Ridge, 45.9962°N, 66.8781°W, 3-18.VII.2014, 30.VI-16.VII.2015, 16-29.VII.2015, C. Alderson & V. Webster // Mixed forest, Lindgren (black) funnel trap in canopy (1), purple Lindgren funnel traps 1 m high (3), black Lindgren funnel trap 1 m high (1) (2, AFC; 3, RWC).

####### Distribution in Canada and Alaska.

ON, QC, **NB** (Bousquet et al. 2013).

####### Comments.

All specimens of this species were captured in Lindgren funnel traps in hardwood and mixed forests. Eleven of the 15 individuals were captured in the canopy of trees.

#### Subfamily Choraginae Kirby, 1819

##### Tribe Choragini Kirby, 1819

###### Choragus harrisii

Taxon classificationAnimaliaColeopteraAnthribidae

LeConte, 1878

####### Material examined.

**New Brunswick, Restigouche Co.**, Jacquet River Gorge P.N.A. (Protected Natural Area), 47.8257°N, 66.0764°W, 22.VII-5.VIII.2014, C. Alderson & V. Webster // Old *Populus balsamifera* stand near river, Lindgren funnel traps in canopy of *Populus balsamifera* (2, RWC).

####### Distribution in Canada and Alaska.

**NB (New Canadian record).** This is the first record of *Choragus harrisii* LeConte for Canada. In the USA, it has been reported from MA west to MI and OK (Valentine 1998).

###### Choragus zimmermanni

Taxon classificationAnimaliaColeopteraAnthribidae

LeConte, 1878

####### Material examined.

**New Brunswick, Queens Co.**, C.F.B. Gagetown, 45.7516°N, 66.1866°W, 17-30.VII.2015, C. Alderson & V. Webster // Old mixed forest with *Quercus rubra*, Lindgren funnel trap 1 m high under trees (1, RWC). **York Co.**, Keswick Ridge, 45.9962°N, 66.8781°W, 13-27.VIII.2015, C. Alderson & V. Webster // Mixed forest, Lindgren funnel trap 1 m high (1, CMNC).

####### Distribution in Canada and Alaska.

**NB (New Canadian record).** This is the first record of *Choragus zimmermanni* LeConte for Canada. In the USA, it has been reported from MA west to OH and south to FL and TX (Valentine 1998).

### Family Nemonychidae Bedel, 1882

#### Subfamily Cimberidinae Gozis, 1882

##### Tribe Cimberidini Gozis, 1882

###### Cimberis pallipennis

Taxon classificationAnimaliaColeopteraNemonychidae

(Blatchley, 1916)

####### Material examined.

**New Brunswick, Gloucester Co.**, Bathurst, Daly Point Nature Preserve, 47.6392°N, 65.6098°W, 13-28.V.2015, 28.V-15.VI.2015, 15-25.VI.2015, C. Alderson & V. Webster // Mixed forest, green Lindgren funnel traps in canopy of white pine (8, RWC). **Kent Co.**, Kouchibouguac National Park, 46.8072°N, 64.9100°W, 21-27.V.2015, 27.V-12.VI.2015, 12-24.VI.2015, 24.VI-7.VII.2015, C. Alderson & V. Webster // Jack pine forest, Lindgren funnel traps, 1 m high (3, AFC; 2, RWC). **Queens Co.**, C.F.B. Gagetown, 45.7516°N, 66.1866°W, 9-22.V.2013, C. Alderson & V. Webster // Old mixed forest with *Quercus rubra*, Lindgren funnel trap in canopy of *Quercus rubra* (1, RWC). **York Co.**, 15 km W of Tracy, off Rt. 645, 45.6848°N, 66.8821°W, 1-8.VI.2009, R. Webster & M.-A. Giguère // Red pine forest, Lindgren funnel trap (1, AFC).

####### Distribution in Canada and Alaska.

AB, QC, **NB**, NS (Bousquet et al. 2013).

####### Comments.

All specimens of *Cimberis pallipennis* (Blatchley) from NB were captured in Lindgren funnel traps. Eight individuals were caught in traps in the canopy of white pine, *Pinus strobus* L.; others were captured in traps 1 m high in jack pine (*Pinus banksiana* Lamb.) and red pine (*Pinus resinosa* Ait.) stands.

### Family Brentidae Billberg, 1820

#### Subfamily Nanophyinae Gistel, 1848

##### Tribe Nanophyini Gistel, 1848

###### Nanophyes marmoratus marmoratus

Taxon classificationAnimaliaColeopteraBrentidae

(Goeze, 1777)†

####### Material examined.

**New Brunswick, Queens Co.**, Grand Lake Meadows P.N.A. at Rt. 105, 45.8461°N, 66.2061°W, 12.VI.2014, 22.VI.2014, R.P. Webster // Old field near flood plain forest, sweeping (4, RWC). **York Co.**, Keswick Ridge, 45.9962°N, 66.8781°W, 3-18.VI.2015, 18-30.VI.2015, C. Alderson & V. Webster // Hardwood forest, green Lindgren funnel trap in canopy (1), black Lindgren funnel trap 1 m high (1) (2, RWC); Fredericton, Odell Park, 45.9508°N, 66.6723°W, 29.VI-14.VII.2015, C. Alderson & V. Webster // Hardwood stand, Lindgren funnel trap in canopy (1, AFC).

####### Distribution in Canada and Alaska.

MB, ON, QC, **NB** (Bousquet et al. 2013).

####### Comments.

*Nanophyes marmoratus marmoratus* (Goeze) was introduced into North America to control purple loosestrife, *Lythrum salicaria* L. (Anderson 2003).

### Family Brachyceridae Billberg, 1820

#### Subfamily Erirhininae Schönherr, 1825

##### Tribe Erirhinini Schönherr, 1825

###### Procas lecontei

Taxon classificationAnimaliaColeopteraBrachyceridae

Bedel, 1879

####### Material examined.

**New Brunswick, Queens Co.**, Grand Lake Meadows P.N.A., 45.8227°N, 66.1209°W, 12.IV-3.VI.2011, M. Roy & V. Webster // Old silver maple forest & seasonally flooded marsh, Lindgren funnel trap (1, RWC).

####### Distribution in Canada and Alaska.

YT, NT, MB, ON, QC, **NB** (Bousquet et al. 2013).

####### Comments.

*Procas lecontei* Bedel is a rarely collected species about which nothing is known of its plant associations or natural history.

### Family Curculionidae Latrielle, 1802

#### Subfamily Curculioninae Latrielle, 1802

##### Tribe Anthonomini C.G. Thomson, 1859

###### Anthonomus (Anthonomus) pusillus

Taxon classificationAnimaliaColeopteraCurculionidae

LeConte, 1876

####### Material examined.

**New Brunswick, Gloucester Co.**, Bathurst, Daly Point Nature Preserve, 47.6392°N, 65.6098°W, 9-23.VII.2015, C. Alderson & V. Webster // Mixed forest, green Lindgren funnel trap 1 m high (1, RWC).

####### Distribution in Canada and Alaska.

**NB (New Canadian record).**

####### Comments.

*Anthonomus pusillus* LeConte has been recorded as far north as MA, NY, and NJ in the USA; it is associated with common frostweed, *Crocanthemum canadense* (L.) Britten (Cistaceae) (Blatchley and Leng 1916), which has been recorded from NS and PE but has not yet been found in NB. It is likely that this plant, which lives in dry sandy areas with thin tree cover, will be found in NB. Sandy dune-like areas occur in the vicinty of the site where *Anthonomus pusillus* was found. Other members of the Cistaceae (*Hudsonia tomentosa* Nutt., *Lechea maritima* Legget *ex* BSP) have been recorded near this locality (Hinds 2000).

###### Anthonomus (Cnemocyllus) pictus

Taxon classificationAnimaliaColeopteraCurculionidae

Blatchley, 1922

####### Material examined.

**New Brunswick, Sunbury Co.**, Maugerville, off Rt. 105, 45.8662°N, 66.4559°W, 9.VI.2014, R.P. Webster // Flood plain forest, sweeping roadside foliage (1, RWC).

####### Distribution in Canada and Alaska.

ON, QC, **NB**, NS (Bousquet et al. 2013).

####### Comments.

Specimen labels as “in gall on goldenrod” are the only indications of plant associations for this species (Clark and Burke 2005).

##### Tribe Curculionini Latreille, 1802

###### Subtribe Archariina Pelsue & O’Brien, 2011

####### Archarius salicivorus

Taxon classificationAnimaliaColeopteraCurculionidae

(Paykull, 1792)†

######## Material examined.

**New Brunswick, Sunbury Co.**, Maugerville, off Rt. 105, 45.8662°N, 66.4559°W, 4.VI.2014, R.P. Webster // Flood plain forest, sweeping roadside foliage (1, RWC).

######## Distribution in Canada and Alaska.

QC, **NB** (Bousquet et al. 2013).

######## Comments.

This species is associated with galls on *Salix* (Salicaecae) (Anderson 2002).

##### Tribe Ellescini C.G. Thomson, 1859

###### Subtribe Dorytomina Bedel, 1886

####### Dorytomus hirtus

Taxon classificationAnimaliaColeopteraCurculionidae

LeConte, 1876

######## Material examined.

**New Brunswick, Restigouche Co.**, Jacquet River Gorge P.N.A., 47.8257°N, 66.0764°W, 15-29.V.2014, C. Alderson & V. Webster // Old *Populus balsamifera* stand near river, Lindgren funnel traps in canopy of *Populus balsamifera* (2, AFC; 1, NBM; 11, RWC); ca. 3 km SE of Simpsons Field, 47.5277°N, 66.5142°W, 14-28.V.2015, C. Alderson & V. Webster // Old cedar & spruce forest with *Populus balsamifera* & *Populus tremuloides*, Lindgren funnel trap in canopy of *Populus balsamifera* (1, AFC).

######## Distribution in Canada and Alaska.

YT, BC, AB, **NB** (Bousquet et al. 2013). These are the first eastern records of *Dorytomus hirtus* LeConte. Previously, this species was known as far east as AB in Canada and IN in the USA (O’Brien 1970, Bousquet et al. 2013). This species will undoubtedly be found in the intervening areas and is likely transcontinental in Canada.

######## Comments.

In the western areas of its range, *Dorytomus hirtus* is associated with *Populus fremonti* S. Watson (O’Brien 1970). In NB, this species was captured in Lindgren funnel traps in balsam poplar, *Populus balsamifera* L., the probable host in this region. All (15) specimens of *Dorytomus hirtus* were captured in Lindgren funnel traps in the canopy of *Populus balsamifera*, none in traps in the understory. Adults and larvae of this genus are associated with reproductive structures of various Salicaceae.

####### Subtribe Ellescina

Taxon classificationAnimaliaColeopteraCurculionidae

C.G. Thomson, 1859

######## Comments.

Specimens reported by Webster et al. (2012) as *Ellescus ephippiatus* (Say) were misidentified and were *Ellescus bipunctatus* (Linnaeus), a new provincial record. One specimen of *Ellescus ephippiatus* was collected in NB, thus maintaining this species on the provincial list. Adults and larvae of this genus are associated with reproductive structures of various Salicaceae. Species are very poorly defined, and the genus needs revision.

####### Ellescus bipunctatus

Taxon classificationAnimaliaColeopteraCurculionidae

(Linnaeus, 1758)

######## Material examined.

**New Brunswick, Carleton Co.**, Meduxnekeag Valley Nature Preserve, 46.1878°N, 67.6705°W, 18.VIII.2008, R.P. Webster // Hardwood forest, sweeping (1, NBM); Meduxnekeag Valley Nature Preserve, 46.1907°N, 67.6740°W, 8-23.V.2012, C. Alderson & V. Webster // Old mixed forest, Lindgren funnel traps in canopy of *Populus tremuloides* (2, RWC). **Queens Co.**, Cranberry Lake P.N.A., 46.1125°N, 65.6075°W, 3-13.V.2011, 13-25.V.2011, M. Roy & V. Webster // old red oak forest, Lindgren funnel traps in forest canopy (trap in big toothed aspen) (5, AFC; 2, NBM; 8, RWC); C.F.B. Gagetown, 45.7516°N, 66.1866°W, 9-22.V.2013, 20.V-4.VI.2015, C. Alderson & V. Webster // Old mixed forest with *Quercus rubra*, Lindgren funnel traps in canopy (1, AFC; 1 RWC). **Northumberland Co.**, ca. 1.5 km NW of Sevogle, 47.0939°N, 65.8387°W, 1-14.V..2013, C. Alderson & V. Webster // *Populus tremuloides* stand with a few conifers, Lindgren funnel trap in canopy of *Populus tremuloides* (1, AFC). **York Co.**, Fredericton, 12.V.1921, 19.V.1921, 10.V.1921, R.P.G. (16, AFC); Fredericton, Odell Park, 45.9539°N, 66.6666°W, 2-15.V.2013, C. Alderson & V. Webster // Hardwood stand, Lindgren funnel trap in canopy (1, AFC); Keswick Ridge, 45.9962°N, 66.8781°W, 22.V-4.VI.2014, C. Alderson & V. Webster // Hardwood forest, Lindgren funnel trap (1, AFC).

######## Distribution in Canada and Alaska.

MB, ON, QC, **NB** (Bousquet et al. 2013).

######## Comments.

Many (21) specimens of *Ellescus bipunctatus* were captured in Lindgren funnel traps in hardwood and mixed forests in NB; all but one in the canopy of trees. Most specimens were collected from traps in the canopy of quaking aspen, *Populus tremuloides* Michx. and large-toothed aspen, *Populus grandidentata* Michx.

####### Ellescus ephippiatus

Taxon classificationAnimaliaColeopteraCurculionidae

(Say, 1832)

######## Material examined.

**New Brunswick, Sunbury Co.**, Maugerville, off Rt. 105, 45.8662°N, 66.4559°W, 4.VI.2014, R.P. Webster // Flood plain forest, sweeping roadside foliage (1, RWC).

######## Distribution in Canada and Alaska.

YT, BC, AB, SK, MB, ON, QC, NB (Bousquet et al. 2013).

##### Tribe Mecinini Gistel, 1848

###### Mecinus janthinus

Taxon classificationAnimaliaColeopteraCurculionidae

(Germar, 1821)†

####### Material examined.

**New Brunswick, Queens Co.**, Grand Lake Meadows P.N.A., off Rt. 105, 45.8461°N, 66.2061°W, 12.VI.2014, R.P. Webster // Old field near flood-plain forest, sweeping (1, NBM). **Sunbury Co.**, Maugerville, off Rt. 105, 45.8662°N, 66.4559°W, 4.VI.2014, 9.VI.2014, R.P. Webster // Flood plain forest, sweeping roadside foliage (2, AFC; 2, NBM; 10, RWC). **York Co.**, Douglas, Currie Mountain, 45.9844°N, 66.7592°W, 3-15.V.2013, C. Alderson & V. Webster // Mixed forest with *Quercus rubra*, Lindgren funnel trap 1 m high under *Quercus rubra* (1, RWC).

####### Distribution in Canada and Alaska.

BC, AB QC, **NB**, NS (Bousquet et al. 2013).

####### Comments.

This species was introduced into North America for the control of yellow and Dalmatian toadflax, *Linaria vulgaris* (L.) Mill. and *Linaria dalmatica* (L.) Mill. (Scrophulariaceae) (Jeanneret and Schroeder 1992).

##### Tribe Otidocephalini Lacordaire, 1863

###### Myrmex chevrolatii

Taxon classificationAnimaliaColeopteraCurculionidae

(Horn, 1873)

####### Material examined.

**New Brunswick, York Co.**, Douglas, Currie Mountain, 45.9832°N, 66.7564°W, 24.VI-9.VII.2013, C. Alderson & V. Webster // Old *Pinus strobus* stand, Lindgren funnel traps in canopy of *Pinus strobus* (1, AFC; 1, RWC); Douglas, Currie Mountain, 45.9844°N, 66.7592°W, 24.VI-9.VII.2013, 9-24.VII.2013, 24.VII-7.VIII.2013, C. Alderson & V. Webster // Mixed forest with *Quercus rubra*, Lindgren funnel traps in canopy of *Quercus rubra* (7, AFC; 1, NBM; 10, RWC).

####### Distribution in Canada and Alaska.

ON, QC, **NB** (Bousquet et al. 2013).

####### Comments.

All (20) specimens of *Myrmex chevrolati* (Horn) were captured in Lindgren funnel traps in the canopy of trees (mostly in red oak (*Quercus rubra* L.)), none in traps in the understory. Adults in the Canadian Museum of Nature collection from TX have been associated with *Smilax* (Smilacaceae) vines.

##### Tribe Rhamphini Rafinesque, 1815

###### Orchestes alni

Taxon classificationAnimaliaColeopteraCurculionidae

(Linnaeus, 1758)†

####### Material examined.

**Nova Scotia, Halifax Co.**, Magazine Hill, 44.4285°N, 63.3798°W, 14.V.2015, K. Van Rooyen & M. Luco // DND2 Black 2, monochamol lure, High trap – 4 funnel Lindgren (1, AFC).

####### Distribution in Canada and Alaska.

BC, ON, QC, **NS** (Bousquet et al. 2013). This adventive European species, associated with *Ulmus*, was first reported from western North America by Anderson et al. (2007) and has since become widespread (Looney et al. 2012, Douglas et al. 2013). The above record is the first report of this species from the Maritime Provinces.

#### Subfamily Baridinae Schönherr, 1836

##### Tribe Madarini Jekel, 1865

###### Subtribe Madarina Jekel, 1865

####### Madarellus undulatus

Taxon classificationAnimaliaColeopteraCurculionidae

(Say, 1824)

######## Material examined.

**New Brunswick, Kent Co.**, Kouchibouguac National Park, 46.8072°N, 64.9100°W, 12-24.VI.2015, C. Alderson & V. Webster // Jackpine forest, Lindgren funnel trap, 1 m high (1, RWC). **York Co.**, Keswick Ridge, 45.9962°N, 66.8781°W, 19.V-3.VI.2015, 3-18.VI.2015, C. Alderson & V. Webster // Hardwood forest, black Lindgren funnel traps 1 m high (4), green Lindgren funnel trap 1 m high (1) (5, RWC).

######## Distribution in Canada and Alaska.

ON, QC, **NB**, NS (Bousquet et al. 2013).

######## Comments.

All specimens of this species were captured in Lindgren funnel traps. The species is associated with wild grape, *Vitis* and Virginia creeper, *Parthenocissus* (both Vitaceae) (Blatchley and Leng 1916).

#### Subfamily Ceutorhynchinae Gistel, 1848

##### Tribe Ceutorhynchini Gistel, 1848

###### Microplontus campestris

Taxon classificationAnimaliaColeopteraCurculionidae

(Gyllenhal, 1837)†

####### Material examined.

**New Brunswick, Westmorland Co.**, Rt. 15 at exit 53, 45.2079°N, 64.3085°W, 17.VI.2014, M.-A. Giguère & R.P. Webster // Roadside, sweeping (2, RWC).

####### Distribution in Canada and Alaska.

ON, QC, **NB** (Bousquet et al. 2013).

####### Comments.

This species is associated with *Chrysanthemum leucanthemum* L. (Asteraceae) (Anderson 2002).

##### Tribe Mononychini LeConte, 1876

###### Mononychus vulpeculus

Taxon classificationAnimaliaColeopteraCurculionidae

(Fabricius, 1801)

####### Comments.

Bousquet et al. (2013) did not not include *Mononychus vulpeculus* (Fabricius) as occurring in NB in their checklist. Majka et al. (2007) reported this species from several sites in the province, where it is often found on *Iris versicolor* L. This species should therefore be included on the faunal list of NB.

##### Tribe Phytobiini Gistel, 1848

###### Pelenomus waltoni

Taxon classificationAnimaliaColeopteraCurculionidae

(Boheman, 1843)†

####### Material examined.

**New Brunswick, Gloucester Co.**, Bathurst, Daly Point Nature Preserve, 47.6392°N, 65.6098°W, 13-28.V.2015, C. Alderson & V. Webster // Mixed forest, black Lindgren funnel trap in canopy (1, RWC). **York Co.**, Keswick Ridge, 45.9962°N, 66.8781°W, 19.V-3.VI.2015, 3-18.VI.2015, C. Alderson & V. Webster // Hardwood forest, black Lindgren funnel traps 1 m high under trees (2), green Lindgren funnel traps 1 m high under trees (3), purple Lindgren funnel trap 1 m high under trees (1), (1, CMNC; 5, RWC).

####### Distribution in Canada and Alaska.

ON, QC, **NB** (Bousquet et al. 2013).

####### Comments.

All specimens of this adventive species were captured in Lindgren funnel traps. In Europe, this species has been associated with *Polygonum hydropiper* L. and *Polygonum mite* Schrank (Polygonaceae) (Anderson and Korotyaev 2004); host associations in North America are likely also with *Polygonum* species.

###### Rhinoncus bruchoides

Taxon classificationAnimaliaColeopteraCurculionidae

(Herbst, 1784)†

####### Material examined.

**New Brunswick, Carleton Co.**, Wilmot, Two Mile Brook Fen, Wakefield, 46.3594°N, 67.6800°W, 2.VI.2005, R.P. Webster, coll. // On trail through cedar swamp, in flight in late afternoon (1, NBM); Meduxnekeag Valley Nature Preserve, 46.1890°N, 67.6766°W, 4.VII.2005, M.-A. Giguère & R.P. Webster, coll. // Flood plain forest, with butternut, sweeping (1, RWC); Florenceville, 46.4613°N, 67.6239°W, 16.VI.2010, M.-A. Giguère // Potato field, yellow pan trap (1, RWC). **Queens Co.**, W. of Jemseg at “Trout Creek”, 45.8237°N, 66.1225°W, 6.IX.2007, R.P. Webster, coll. // Silver maple swamp, sweeping foliage, along margin of marsh (1, RWC). **Restigouche Co.**, Jacquet River Gorge P.N.A., 47.8257°N, 66.0764°W, 29.V-10.VI.2014, C. Alderson & V. Webster // Old *Populus balsamifera* stand near river, Lindgren funnel trap in canopy of *Populus balsamifera* (1, RWC). **Sunbury Co.**, Gilbert Island, 45.8770°N, 66.2954°W, 18-28.V.2012, 28.V-12.VI.2012, C. Alderson, C. Hughes, & V. Webster // hardwood forest, Lindgren funnel traps in canopy of *Juglans cinerea*, & 1 m high under *Juglans cinerea* (2, RWC). **York Co.**, Lincoln, Agriculture Canada Exp. Farm, 13.VI.2012, M.-A. Giguère // Potato field, yellow pan trap (1, RWC).

####### Distribution in Canada and Alaska.

QC, **NB**, NS (Bousquet et al. 2013).

####### Comments.

*Rhinoncus bruchoides* (Herbst) has been associated with a number of species of *Polygonum* (Polygonaceae) (Hoebeke and Whitehead 1980).

###### Rhinoncus perpendicularis

Taxon classificationAnimaliaColeopteraCurculionidae

(Reich, 1797)†

####### Material examined.

**New Brunswick, Queens Co.**, Grand Lake Meadows P.N.A. at Rt. 105, 45.8461°N, 66.2061°W, 22.VI.2014, R.P. Webster // Old field near flood plain forest, sweeping (1, CMNC). **York Co.**, Keswick Ridge, 45.9962°N, 66.8781°W, 22.V-4.VI.2014, C. Alderson & V. Webster // Field/meadow, Lindgren funnel trap (1, RWC).

####### Distribution in Canada and Alaska.

ON, QC, **NB** (Bousquet et al. 2013).

####### Comments.

In ON, this species has been associated with *Polygonum hydropiper* L. (Polygonaceae) (Anderson and Korotyaev 2004).

###### *Rhinoncus pericaripius* (Linnaeus, 1758)† and *Rhinoncus castor* (Fabricius, 1792)†

The names for these two species of *Rhinoncus* (both recorded from NB) have recently been changed based on examination of type specimens (Huang and Colonnelli 2014). The species formerly known as *Rhinoncus castor* (Fabricius, 1792) is now known as *Rhinoncus pericarpius* (Linnaeus, 1758). Following this change, the species formerly known as *Rhinoncus pericarpius* (Linnaeus, 1758) is now known as *Rhinoncus leucostigma* (Marsham, 1802).

#### Subfamily Cossoninae Schönherr, 1825

##### Tribe Cossonini Schönherr, 1825

###### Cossonus impressifrons

Taxon classificationAnimaliaColeopteraCurculionidae

Boheman, 1838

####### Material examined.

**New Brunswick, Restigouche Co.**, Jacquet River Gorge P.N.A., 47.8257°N, 66.0764°W, 29.V-10.VI.2014, 10-25.VI.2014, C. Alderson & V. Webster // Old *Populus balsamifera* stand near river, Lindgren funnel traps 1 m high under trees (8) in canopy of *Populus balsamifera* (3) (3, AFC; 8, RWC); ca. 3 km SE of Simpsons Field, 47.5277°N, 66.5142°W, 25.VI-10.VII.2015, 10-23.VII.2015, C. Alderson & V. Webster // Old cedar & spruce forest with *Populus balsamifera* & *Populus tremuloides*, Lindgren funnel traps in canopy of *Populus balsamifera* (1, AFC; 3, RWC).

####### Distribution in Canada and Alaska.

ON, QC, **NB** (Bousquet et al. 2013).

####### Comments.

All individuals of *Cossonus impressifrons* Boheman were captured in Lindgren funnel traps in areas with *Populus balsamifera*, either under these trees or in the canopy.

###### Cossonus pacificus

Taxon classificationAnimaliaColeopteraCurculionidae

Van Dyke, 1916

####### Material examined.

**New Brunswick, Northumberland Co.**, ca. 1.5 km NW of Sevogle, 47.0939°N, 65.8387°W, 11-26.VI.2013, 26.VI-8.VII.2013, C. Alderson & V. Webster // *Populus tremuloides* stand with a few conifers, Lindgren funnel traps in canopy of *Populus tremuloides* (2, RWC).

####### Distribution in Canada and Alaska.

BC, AB, SK, **NB** (Bousquet et al. 2013). This is the first eastern record of this species. Previously, it was known as far east as SK but is probably more widespread.

##### Tribe Rhyncolini Gistel, 1848

###### Subtribe Rhyncolina Gistel, 1848

####### Rhyncolus knowltoni

Taxon classificationAnimaliaColeopteraCurculionidae

(Thatcher, 1940)

######## Material examined.

**Charlotte Co.**, St. Andrews, 45.0741°N, 67.0383°W, 22.VII.2012, R.P. Webster // Barrier beach (gravel), under large log (1, CMNC; 1, RWC).

######## Distribution in Canada and Alaska.

AB, SK, MB, **NB** (Bousquet et al. 2013). This is the first eastern record of this species. Previously, it was known from MB with additional records added from AB and SK by Douglas et al. (2013). *Rhyncolus knowltoni* (Thatcher) is associated with *Populus tremuloides* (Douglas et al. (2013) and is undoubtedly more widespread than the records indicate.

#### Subfamily Cryptorhynchinae Schönherr, 1825

##### Tribe Cryptorhynchini Schönherr, 1825

###### Subtribe Cryptorhynchina Schönherr, 1825

####### Eubulus bisignatus

Taxon classificationAnimaliaColeopteraCurculionidae

(Say, 1832)

######## Material examined.

**New Brunswick, Northumberland Co.**, ca. 1.5 km NW of Sevogle, 47.0939°N, 65.8387°W, 11-26.VI.2013, C. Alderson & V. Webster // *Populus tremuloides* stand with a few conifers, Lindgren funnel trap in canopy of *Populus tremuloides* (1, RWC). **Queens Co.**, C.F.B. Gagetown, 45.7516°N, 66.1866°W, 4-17.VI.2013, 17.VI-3.VII.2013, C. Alderson & V. Webster // Old mixed forest with *Quercus rubra*, Lindgren funnel traps in canopy of *Populus grandifolia* (1, AFC; 3, RWC). **Sunbury Co.**, Gilbert Island, 45.8770°N, 66.2954°W, 20.VI-5.VII.2013, C. Alderson, C. Hughes, & V. Webster // hardwood forest, Lindgren funnel traps in canopy of *Populus tremuloides* (2, RWC). **York Co.**, Keswick Ridge, 45.9962°N, 66.8781°W, 4-19.VI.2014, 19.VI-3.VII.2014, 3-18.VII.2014, C. Alderson & V. Webster // Mixed forest, Lindgren funnel traps in canopy (2, AFC; 1, NBM; 5, RWC); Fredericton, Odell Park, 45.9508°N, 66.6723°W, 1-19.V.2015, C. Alderson & V. Webster // Hardwood stand, Lindgren funnel trap in canopy *Populus tremuloides* (1, AFC).

######## Distribution in Canada and Alaska.

ON, QC, **NB** (Bousquet et al. 2013).

######## Comments.

All 16 specimens of *Eubulus bisignatus* (Say) were captured in Lindgren funnel traps in the canopy of either *Populus tremuloides* or *Populus grandifolia*; none in traps in the understory. The species is most often collected in light traps and has been associated with a variety of hardwood trees (Anderson 2008).

#### Subfamily Entiminae Schönherr, 1823

##### Tribe Polydrusini Schönherr, 1823

###### Polydrusus cervinus

Taxon classificationAnimaliaColeopteraCurculionidae

(Linnaeus, 1758)†

####### Material examined.

**New Brunswick, Kent Co.**, Kouchibouguac National Park, 46.8072°N, 64.9100°W, 27.V-12.VI.2015, C. Alderson & V. Webster // Jackpine forest, Lindgren funnel trap, 1 m high (1, AFC). **Gloucester Co.**, Bathurst, Daly Point Nature Preserve, 47.6392°N, 65.6098°W, 9-23.VII.2015, 5-21.VIII.2015, C. Alderson & V. Webster // Mixed forest, purple Lindgren funnel traps 1 m high (2, AFC). **Queens Co.**, Rt. 690 near Flowers Cove, 46.0367°N, 66.0376°W, 16.VI.2013, 20.VI.2013, M. Giguère & R. Webster // Roadside near stand of *Robinia pseudoacacia*, beating *Robinia* foliage (1, AFC; 1, RWC); C.F.B. Gagetown, 45.7516°N, 66.1866°W, 4-17.VI.2013, C. Alderson & V. Webster // Old mixed forest with *Quercus rubra*, Lindgren funnel traps in canopy of *Quercus rubra* (1, AFC). **Sunbury Co.**, 9.5 km NE jct. 101 & 645, 45.7586°N, 66.6755°W, 2.VII.2008, R.P. Webster // Old field with open sandy areas, on *Salix* sp. (2, RWC). **York Co.**, Charters Settlement, 45.8286°N, 66.7365°W, 8.VII.2005, R.P. Webster // Mixed forest, on foliage of *Salix* sp. (1, RWC); Charters Settlement, 45.8430°N, 66.7275°W, 11.VII.2005, R.P. Webster // Regenerating forest, beating foliage (1, RWC); same locality data, collector, and forest type but 12.VII.2005 // On foliage of *Salix* sp. (1, RWC); same locality data and collector but 21.VI.2008 // Regenerating forest, brushy opening, sweeping foliage (1 RWC); Fredericton, 45.9154°N, 66.6687°W, 30.V.2010, R.P. Webster // Roadside, on *Salix* sp. (2, RWC); Douglas, Currie Mountain, 45.9844°N, 66.7592°W, 10-24.VI.2013, C. Alderson & V. Webster // Mixed forest with *Quercus rubra*, Lindgren funnel trap in canopy of *Quercus rubra* (1), 1 m high under *Quercus rubra* (1, AFC; 1, RWC); same locality and collectors but 45.9832°N, 66.7564°W, 27.V-10.VI.2013, 24.VI-9.VII.2013 // Old *Pinus strobus* stand, Lindgren funnel trap in canopy of *Pinus strobus* (2, AFC); Fredericton, Odell Park, 45.9539°N, 66.6666°W, 10-24.VI.2013, C. Alderson & V. Webster // Hardwood stand, Lindgren funnel trap in canopy (2, AFC); Keswick Ridge, 45.9962°N, 66.8781°W, 19.VI-3.VII.2014, C. Alderson & V. Webster // Mixed forest, Lindgren funnel trap 1 m high under trees (1, AFC).

####### Distribution in Canada and Alaska.

QC, **NB**, NS, PE (Bousquet et al. 2013).

####### Comments.

This adventive Palaearctic species is widespread in NB. A few adults were found on *Salix* foliage, but most NB specimens were captured in Lindgren funnel traps in various forest types.

###### Polydrusus impressifrons

Taxon classificationAnimaliaColeopteraCurculionidae

Gyllenhal, 1834†

####### Comments.

*Polydrusus impressifrons* Gyllenhal was newly reported from NB by Majka et al. (2007), based on records from Charters Settlement and Moncton (in the University of Moncton Insect Collection). The specimens from Charters Settlement were misidentified and are *Polydrusus cervinus* (Linnaeus); however, the specimen from Moncton was correctly determined and is *Polydrusus impressifrons*, and the species remains on the provincial list.

#### Subfamily Mesoptiliinae Lacordaire, 1863

##### Tribe Magdalidini Pascoe, 1870

###### Magdalis piceae

Taxon classificationAnimaliaColeopteraCurculionidae

Buchanan, 1934

####### Material examined.

**New Brunswick, Northumberland Co.**, Upper Graham Plains, 47.1001°N, 66.8154°W, 9-24.VII.2014, C. Alderson & V. Webster // Old black spruce forest, Lindgren funnel trap (2, RWC). **Sunbury Co.**, Acadia Research Forest, 45.9866°N, 66.3841°W, 4-11.VIII.2009, R. Webster & M.-A. Giguère, coll. // Red spruce forest with red maple & balsam fir, Lindgren funnel trap (1, RWC). **York Co.**, 16 km W of Tracy off Rt. 645, 45.6854°N, 66.8839°W, 11-25.VII.2014, C. Alderson & V. Webster // Old red pine forest, Lindgren funnel trap (1, RWC); Fredericton, Odell Park, 45.9508°N, 66.6723°W, 29.VI-14.VII.2015, 25.VIII-9.IX.2015, C. Alderson & V. Webster // Hardwood stand, Lindgren funnel traps in canopy (3), 1 m high under trees (1) (1, CMNC; 3, RWC).

####### Distribution in Canada and Alaska.

ON, QC, **NB**, NS, PE (Bousquet et al. 2013).

####### Comments.

All specimens of this species were captured in Lindgren funnel traps. This species was found in an old black spruce (*Picea mariana* (Mill.) B.S.P.) forest, a red spruce (*Picea rubens* Sarg.) forest with red maple (*Acer rubrum* L.) and balsam fir (*Abies balsamea* (L.) Mill.), an old red pine forest, and in a hardwood stand with spruce nearby.

#### Subfamily Scolytinae Latreille, 1804

##### Tribe Cryphalini Lindemann, 1877

###### Procryphalus mucronatus

Taxon classificationAnimaliaColeopteraCurculionidae

(LeConte, 1879)

####### Material examined.

**New Brunswick, Carleton Co.**, Meduxnekeag Valley Nature Preserve, 46.1907°N, 67.6740°W, 7-21.VI.2012, C. Alderson & V. Webster // Old mixed forest, Lindgren funnel traps in canopy of *Populus tremuloides* (6) and 1 m high under *Populus tremuloides* (1) (7, RWC). **Kent Co.**, Kouchibouguac National Park, 46.8087°N, 64.9078°W, 27.V-12.VI.2015, C. Alderson & V. Webster // Poplar/red maple stand, Lindgren funnel trap, 1 m high (1, AFC). **Restigouche Co.**, ca. 3 km SE of Simpsons Field, 47.5277°N, 66.5142°W, 25.VI-10.VII.2015, C. Alderson & V. Webster // Old cedar & spruce forest with *Populus balsamifera* & *Populus tremuloides*, Lindgren funnel trap in canopy of *Populus balsamifera* (1, RWC). **Sunbury Co.**, Gilbert Island, 45.8770°N, 66.2954°W, 18-28.V.2012, 28.V-12.VI.2012, 23.V-6.VI.2013, C. Alderson, C. Hughes, & V. Webster // hardwood forest, Lindgren funnel traps in canopy of *Populus tremuloides* (3, AFC; 3, RWC).

####### Distribution in Canada and Alaska.

AK, BC, AB, **NB** (Bousquet et al. 2013). These are the first records of this species from eastern Canada.

####### Comments.

Most (13 of the 15 specimens) were captured in Lindgren funnel traps in the canopy of *Populus tremuloides* (12) and *Populus balsamifera* (1); the other two individuals were captured in traps under *Populus tremuloides* and in a stand with this tree species present. *Populus tremuloides* is the host of this beetle (Bright 1976). Bright suggested that the record of *Procryphalus utahensis* Hopkins from QC might be a misidentification of *Procryphalus mucronatus* (Bright, personal communication).

##### Tribe Ipini Bedel, 1888

###### Ips grandicollis

Taxon classificationAnimaliaColeopteraCurculionidae

(Eichhoff, 1868)

####### Material examined.

**New Brunswick, Gloucester Co.**, Bathurst, Daly Point Nature Preserve, 47.6392°N, 65.6098°W, 28.V-15.VI.2015, 15-25.VI.2015, 5-21.VIII.2015, C. Alderson & V. Webster // Mixed forest, purple Lindgren funnel traps in canopy of white pine (4, AFC). **Kent Co.**, Kouchibouguac National Park, 46.8072°N, 64.9100°W, 12-24.VI.2015, 4-20.VIII.2015, C. Alderson & V. Webster // Jackpine forest, Lindgren funnel traps, 1 m high (2, AFC). **Northumberland Co.**, ca, 2.5 km W of Sevogle, 47.0876°N, 65.8613°W, 1-14.V.2014, 28.V.-11.VI.2013, 26.VI-8.VII.2013, C. Alderson & V. Webster // Old *Pinus banksiana* stand, Lindgren funnel traps (5, AFC; 2, RWC); Upper Graham Plains, 47.1001°N, 66.8154°W, 4-18.IX.2014, C. Alderson & V. Webster // Old black spruce forest, Lindgren funnel trap (1, AFC). **Queens Co.**, C.F.B. Gagetown, 45.7516°N, 66.1866°W, 20.V-4.VI.2015, C. Alderson & V. Webster // Old mixed forest with *Quercus rubra*, Lindgren funnel traps in canopy (3, AFC). **Restigouche Co.**, Jacquet River Gorge P.N.A., 47.8257°N, 66.0764°W, 29.V-10.VI.2014, C. Alderson & V. Webster // Old *Populus balsamifera* stand near river, Lindgren funnel trap in canopy of *Populus balsamifera* (1, NBM). **Sunbury Co.**, Acadia Research Forest, 45.9990°N, 66.2623°W, 113-26.VI.2012, C. Alderson & V. Webster // Mature balsam fir forest with scattered red spruce & red maple, Lindgren funnel trap (1, RWC). **York Co.**, Douglas, Currie Mountain, 45.9832°N, 66.7564°W, 10-24.VI.2013, 24.VI-9.VII.2013, 24.VII-7.VIII.2013, 7-19.VIII.2013, C. Alderson & V. Webster // Old *Pinus strobus* stand, Lindgren funnel traps in canopy of *Pinus strobus* (13, AFC; 1, NBM; 8, RWC); 16 km W of Tracy off Rt. 645, 45.6854°N, 66.8839°W, 14-26.V.2014, 26.V-9.VI.2014, 23.VI-11.VII.2014, 11-25.VII.2014, C. Alderson & V. Webster // Old red pine forest, Lindgren funnel traps in canopy of red pine (9), 1 m high under trees (1) (8, AFC; 2, NBM); Fredericton, Odell Park, 45.9484°N, 66.6802°W, 22.V-4.VI.2014, 17.VI-3.VII.2014, 17.VII-1.VIII.2014, C. Alderson & V. Webster // Old mixed forest, Lindgren funnel traps in canopy of conifer (2, AFC; 1, NBM).

####### Distribution in Canada and Alaska.

MB, ON, QC, **NB**, NS (Bousquet et al. 2013).

####### Comments.

Most (42 of 54) specimens of *Ips grandicollis* (Eichhoff) were captured in Lindgren funnel traps in the canopy of trees (many other individuals were not vouchered). Specimens were captured in the canopy of eastern white pine (26), balsam fir (1), and red pine (9). The other individuals were captured in stands with white pine and jack pine present. White pine was the only pine present at the Bathurst and Currie Mountain sites, indicating that white pine may be a host for *Ips grandicollis* in NB.

##### Tribe Xyleborini LeConte, 1876

###### Xyleborinus attenuatus

Taxon classificationAnimaliaColeopteraCurculionidae

(Blandford, 1894)†

####### Material examined.

**New Brunswick, Gloucester Co.**, Bathurst, Daly Point Nature Preserve, 47.6392°N, 65.6098°W, 13-28.V.2015, 28.V-15.VI.2015, 25.VI-9.VII.2015, C. Alderson & V. Webster // Mixed forest, black Lindgren funnel traps in canopy (2), 1 m high under trees (1) (3, AFC). **Queens Co.**, C.F.B. Gagetown, 45.7516°N, 66.1866°W, 22.V-4.VI.2013, C. Alderson & V. Webster // Old mixed forest with *Quercus rubra*, Lindgren funnel trap in canopy of *Quercus rubra* (1, RWC). **Restigouche Co.**, Jacquet River Gorge P.N.A., 47.8257°N, 66.0764°W, 15-29.V.2014, 29.V-10.VI.2014, C. Alderson & V. Webster // Old *Populus balsamifera* stand near river, Lindgren funnel traps in canopy of *Populus balsamifera* (1, AFC; 1, NBM). **York Co.**, Douglas, Currie Mountain, 45.9832°N, 66.7564°W, 3-15.V.2013, C. Alderson & V. Webster // Old *Pinus strobus* stand, Lindgren funnel traps in canopy of *Pinus strobus* (3, RWC); Douglas, Currie Mountain, 45.9844°N, 66.7592°W, 3-15.V.2013, 15-27.V.2013, 27.V-10.VI.2013, C. Alderson & V. Webster // Mixed forest with *Quercus rubra*, Lindgren funnel traps 1 m high under *Quercus rubra* (5, RWC); Fredericton, Odell Park, 45.9539°N, 66.6666°W, 2-15.V.2013, 27.V-10.VI.2013, C. Alderson & V. Webster // Hardwood stand, Lindgren funnel traps in canopy (2, RWC); same locality but 45.9484°N, 66.6802°W, 12-22.V.2014, 22.V-4.VI.2014, C. Alderson & V. Webster // Old mixed forest, Lindgren funnel traps in canopy of conifer (3), in canopy of hardwood (2), 1 m high under trees (5) (10, AFC); 16 km W of Tracy off Rt. 645, 45.6854°N, 66.8839°W, 14-26.V.2014, 26.V-9.VI.2014, 23.VI-11.VII.2014, 11-25.VII.2014, C. Alderson & V. Webster // Old red pine forest, Lindgren funnel traps in canopy of red pine (2), 1 m high under trees (2) (2, AFC; 2, NBM); Keswick Ridge, 45.9962°N, 66.8781°W, 6-22.V.2014, C. Alderson & V. Webster // Mixed forest, Lindgren funnel trap 1 m high under trees (1, AFC); Canterbury, Eel River P.N.A., 45.8966°N, 67.6345°W, 8-21.V.2014, C. Alderson & V. Webster // Old-growth eastern white cedar swamp & fen, Lindgren funnel traps (1, AFC; 1, NBM).

####### Distribution in Canada and Alaska.

BC, ON, QC, **NB**, NS, PE (Bousquet et al. 2013).

####### Comments.

The adventive *Xyleborinus attenuatus* (Blandford) was first reported from North America by Mudge et al. (2001) from the northwestern USA, followed by reports from BC, the northeastern USA, PE, NS, and QC (Douglas et al. 2013). This species is now widespread and locally abundant in NB (numerous individuals captured in Lindgren funnel traps at Odell Park during 2014 and 2015; only a few were vouchered). All specimens from NB were captured in Lindgren funnel traps, about half in the canopy of trees.

## Supplementary Material

XML Treatment for Eusphyrus walshii

XML Treatment for Choragus harrisii

XML Treatment for Choragus zimmermanni

XML Treatment for Cimberis pallipennis

XML Treatment for Nanophyes marmoratus marmoratus

XML Treatment for Procas lecontei

XML Treatment for Anthonomus (Anthonomus) pusillus

XML Treatment for Anthonomus (Cnemocyllus) pictus

XML Treatment for Archarius salicivorus

XML Treatment for Dorytomus hirtus

XML Treatment for Ellescina

XML Treatment for Ellescus bipunctatus

XML Treatment for Ellescus ephippiatus

XML Treatment for Mecinus janthinus

XML Treatment for Myrmex chevrolatii

XML Treatment for Orchestes alni

XML Treatment for Madarellus undulatus

XML Treatment for Microplontus campestris

XML Treatment for Mononychus vulpeculus

XML Treatment for Pelenomus waltoni

XML Treatment for Rhinoncus bruchoides

XML Treatment for Rhinoncus perpendicularis

XML Treatment for Cossonus impressifrons

XML Treatment for Cossonus pacificus

XML Treatment for Rhyncolus knowltoni

XML Treatment for Eubulus bisignatus

XML Treatment for Polydrusus cervinus

XML Treatment for Polydrusus impressifrons

XML Treatment for Magdalis piceae

XML Treatment for Procryphalus mucronatus

XML Treatment for Ips grandicollis

XML Treatment for Xyleborinus attenuatus
